# Age- and sex-associated differences in Lujo hemorrhagic fever pathogenesis in strain 13/N guinea pigs

**DOI:** 10.1371/journal.pntd.0013633

**Published:** 2025-10-21

**Authors:** Nikesh Tailor, Jérémie Prévost, Geoff Soule, Yvon Deschambault, Angela Sloan, Mable Chan, David Safronetz

**Affiliations:** 1 Special Pathogens Program, National Microbiology Laboratory, Public Health Agency of Canada, Winnipeg, Manitoba, Canada; 2 Department of Medical Microbiology and Infectious Diseases, Max Rady College of Medicine, University of Manitoba, Winnipeg, Manitoba, Canada; University of Texas Medical Branch, UNITED STATES OF AMERICA

## Abstract

Lujo virus (LUJV) is a highly pathogenic zoonotic arenavirus first identified during a 2008 viral hemorrhagic fever (VHF) outbreak in Southern Africa, exhibiting an 80% case fatality rate. Despite its public health significance, LUJV remains poorly understood, with no approved treatments, vaccines, or known reservoir. Existing small animal models have shown limited disease recapitulation, with strain 13/N guinea pigs emerging as a promising model for LUJV pathogenesis. In this study, we evaluate the influence of age and sex on LUJV disease progression in strain 13/N guinea pigs. We show that young females exhibit markedly improved survival, while all young males, as well as juvenile and adult animals of both sexes, succumbed to infection. Despite similar high titers of LUJV detected in the lungs, liver, spleen, kidneys, and serum of infected animals, survival outcomes strongly correlated with immune responses rather than viral burden. Adult animals and young males developed more severe clinical signs, heightened hematological and biochemical abnormalities, and pronounced cytokine storms, particularly elevated IL-1β, IL-2, IL-6, and CXCL10 levels. In contrast, young females displayed lower inflammatory cytokine profiles, and attenuated clinical disease. These findings underscore that LUJV pathogenesis in guinea pigs is influenced by host immune responses rather than viral replication alone. Our results provide critical insight into age- and sex-dependent mechanisms of LUJV disease and support the utility of the strain 13/N guinea pig model for future medical countermeasures development.

## Author summary

Lujo virus (LUJV) is a rare but highly lethal Mammarenavirus first recognized in 2008 during a small outbreak of viral hemorrhagic fever in Southern Africa, where four out of five infected people died. LUJV disease is not well understood, and there are currently no vaccines or treatments. Previously, inbred strain 13/N guinea pigs have been described as model for LUJV disease, but until now only older animals had been studied. In this study, we examined how age and sex affect the course of LUJV infection in guinea pigs. We found that male animals of all ages, as well as juvenile and older females, developed severe disease and succumb to infection, while young females often survived despite having similar viral burdens as older animals. The key difference between lethal and non-lethal infection was in the immune response, where young females showed a cytokine response similar to uninfected control animals while animals that developed lethal disease showed stronger and uncontrolled inflammatory responses. These findings suggest that the severity of LUJV disease is linked to the degree of immune activation than to viral levels, and they strengthen the guinea pig as a model for developing vaccines and treatments.

## Introduction

Lujo virus (LUJV, *Mammarenavirus lujoense*) is a highly pathogenic virus that belongs to the *Arenaviridae* family [1]. It was first identified in 2008 during an outbreak of viral hemorrhagic fever (VHF) disease in humans in Zambia and South Africa which involved five confirmed cases [2,3]. The clinical manifestations of LUJV infection resemble those of other VHFs, including fever, malaise, gastrointestinal symptoms, and hemorrhagic manifestations [4,5]. Severe cases can progress rapidly to multiorgan system failure and death. There are currently no specific antiviral treatments or vaccines available to treat or prevent LUJV infections [5]. Although to date, only the original outbreak of LUJV has been documented, due to the 80% fatality rate (4/5 cases) and potential for causing severe illness in humans, LUJV remains a significant public health concern with the potential for global implications [5]. Notably, although LUJV is considered a zoonotic virus, the natural reservoir has not been identified, which impedes critical surveillance studies required to define its geographic region of endemicity [3].

Disease modelling efforts for LUJV are limited. Unlike Lassa fever caused by Lassa virus, a related Old World mammarenavirus, LUJV disease is not recapitulated in Cynomolgus macaques [6]. Similarly, newborn 2-day-old mice and 14-day-old weaning mice do not succumb to infection with LUJV [7]. However, well aged (1-1.5 year-old) inbred strain 13/N guinea pigs are highly susceptible to LUJV infection. Clinically, the model is characterized by weight loss, increased body temperatures and other non-descript physical signs of disease (ruffled fur, lethargy, discharge) as well as hematological and biochemical abnormalities indicative of VHF-like disease. In adult guinea pigs, infection is lethal in 11–16 days and analysis of organ specimens collected at necropsy confirmed high viral titers in multiple tissues [7].

In the current study, we sought to expand upon the original characterization of the LUJV strain 13/N guinea pig model and examine the effect of age and sex on the development of LUJV disease. By utilizing mixed-sex groups comprising young, juvenile, and adult guinea pigs, our goal was to identify the susceptible age ranges for LUJV infection and lethal disease. Further, we sought to explore immune parameters associated with lethality in these groups. Our results demonstrate that LUJV infection in male strain 13/N guinea pigs is lethal regardless of age. In contrast, only juvenile and adult female animals exhibited severe disease with high mortality, while young female guinea pigs had a high survival rate. Viral loads assessed near the time of lethal disease were similar across all groups, however cytokine scores varied with adult animals exhibiting higher cytokine responses. Combined our data refines the use of strain 13/N guinea pigs as a disease model for LUJV infection and implicates a role of cytokine storm in disease pathogenesis.

## Methods

### Ethics and biosafety

The research outlined in this study was conducted at the National Microbiology Laboratory (NML) of the Public Health Agency of Canada. Experiments were approved by the Animal Care and Use Committee located at the Canadian Science Center for Human and Animal Health and carried out in accordance with the guidelines provided by the Canadian Council on Animal Care. An approved scoring sheet was applied daily to each animal in order to assess clinical condition and to minimize animal suffering. All work with LUJV was performed in the NML’s biosafety level 4 (BSL-4) laboratory. Sample inactivation was performed according to approved institutional standard appropriate procedures.

### Cells and viruses

Vero cells (CCL-81; American Type Culture Collection) were cultured in minimum essential medium (MEM; Thermo Fisher Scientific). Cultures were maintained at 37°C with 5% CO_2_ and supplemented with 10% fetal bovine serum (FBS; Corning), 2 mM L-glutamine (Thermo Fisher Scientific), and 100 U/mL penicillin-streptomycin (Thermo Fisher Scientific).

The LUJV strain used in these studies was isolated from a patient in the 2008 outbreak (kindly provided by Dr. Pierre Rollin, US Centres for Disease Control and Prevention) [7]. Infectious stocks were grown by inoculating Vero CCL-81 cells and collecting supernatant upon observation of cytopathic effect. Debris were removed by centrifugation at 6000 × g for 5 min, after which aliquots were prepared and cryo-preserved for future use. Viral stocks tested for mycoplasma as previously described [8]. Genomic sequences were determined using Illumina-based methods (accession numbers PX353117, PX353118)

### Animals

Strain 13/N guinea pigs (*Cavia porcellus*) were sourced from the in-house breeding colony maintained at the NML. During the course of the experiments, animals had unlimited access to food and water and were checked at least once daily for clinical condition and disease progression. All experimental procedures were conducted on anesthetized guinea pigs (3–5% inhalational isoflurane maintained in medical oxygen). Prior to study enrollment, guinea pigs had a IPTT-300 electronic temperature transponder (Bio Medic Data Systems) subcutaneously implanted into the intra-scapular region.

### Characterization of LUJV infection in strain 13/N guinea pigs

To confirm the findings of Bird et al. [7], an initial pilot study was performed in 4 adult strain 13 guinea pigs (aged 7–9 months, males). Animals were inoculated with 1x10^5^ TCID_50_ units of LUJV by the intraperitoneal (IP) route. Temperatures and weights were monitored daily and animals were euthanized when they reached the pre-determined endpoint. In a parallel study, 8 juvenile guinea pigs (4 males, 4 females, aged 3–6 months) were inoculated with LUJV as above, and serial blood samples were collected every three days to monitor changes in hematology and biochemical parameters. At the time of euthanasia, citrate-treated blood was also collected to assess markers of coagulation.

Following confirmation of the findings from the original description of the model in adult guinea pigs, as well as the observations of lethal disease in juvenile guinea pigs, we compared disease progression in three age groups of guinea pigs defined as young (1–2 months old), juvenile (3–6 months old), and adult (7–10 months old). Each age group consisted of at least 9 male and 9 female animals for a total of 58 guinea pigs. Animals were infected with 1x10^5^ TCID_50_ units of LUJV by the IP route. Post-inoculation, weights and body temperature were monitored regularly. At 12 DPI, 6 or more animals (3 males, 3–6 females) from each age group were randomly selected and exsanguinated and tissues (lung, liver, kidney and spleen) were collected to assess viral titers. The remaining animals were continuously monitored for disease progression and were either euthanized when they reached the pre-determined humane end-point or at 35 DPI, the study end-point.

#### Infectious virus quantification.

For infectious virus assays, the 50% tissue culture infective dose (TCID_50_) was calculated using the Reed and Muench method and expressed as TCID_50_ per gram of sample (TCID_50_/g) [9]. Briefly, harvested tissue samples were weighed and placed in 600µl of minimal essential medium (MEM, HyClone, GE Healthcare Life Sciences, Logan, UT, USA) supplemented with 1% FBS (Gibco, Life Technologies, Grand Island, NY, USA), and 100 U/mL penicillin–streptomycin before being homogenized in a Bead Ruptor Elite Bead Mill Homogenizer (Omni International, Kennesaw, GA, USA) at 4 m/s for 30 seconds. Samples were clarified by centrifugation at 1500 × *g* for 10 min and ten-fold serially diluted in MEM supplemented with 2% heat-inactivated FBS, 2mM L-glutamine, and 100 U/mL penicillin–streptomycin. For each dilution, 100µl was added to 96-well plates of confluent Vero cells in triplicate and incubated for 7 days at 37 °C with 5% CO_2_. Plates were monitored daily and scored for the presence of cytopathic effect.

**Molecular detection of LUJV RNA:** Tissue samples harvested for viral RNA detection were weighed and homogenized in 600μL RLT buffer using a Bead Ruptor Elite Bead Mill Homogenizer (Omni International, Kennesaw, GA, USA) with a stainless steel bead at 4 m/s for 30 seconds. Total RNA from 30mg tissue samples was extracted with the RNeasy Plus Mini kit (Qiagen) according to manufacturer’s instructions. Serum samples were inactivated using AVL buffer (Qiagen), and RNA was extracted using a viral RNA mini-kit (Qiagen). For detection of LUJV RNA, a 20μl reaction was set up using QuantiTect ProbeRT-PCR kit (Qiagen) and with amplification and detection on a QuantStudio Real-Time PCR system (Qiagen) according to the manufacturer’s specifications using previously described forward (5′-GGCCCATGATGACAAGAACTG) and reverse (5′-CCTCACTTTGTAGTGGGTTTCTGAA) primers in conjunction with a double quenched FAM probe (5′-CTACACCCATTGAACTACCTGAGGCTCCTG) (all from IDT) targeting the nucleocapsid (N) gene was used [10]. Thermocycling consisted of a initial reverse transcription step of 50°C for 30 minutes, followed by a 15-minute denaturation at 95°C, followed by 40 cycles of 95 °C for 15 s and 60 °C for 60 s.

**Serological detection of LUJV antibodies:** An in-house enzyme-linked immunosorbent assay (ELISA) based on a recombinant LUJV N antigen was used for the detection of anti-LUJV IgG antibodies in serum collected from experimentally infected guinea pigs. Prior to running the ELISA, guinea pig serum samples were inactivated with gamma irradiation (5mRad, Cobalt-60 source). Individual wells of high-binding 96-well plates (Corning) were coated with 100ng of full length recombinant, purified LUJV N antigen (Biomatik) diluted in 100µl of phosphate-buffered saline (PBS) and stored at 4°C overnight. The following day, plates were washed three times with PBS containing 0.1% Tween 20 (PBS-T) on an automated plate washer (Biotek) and blocked for 1 hr at RT with PBS-T supplemented with 5% skim milk. Plates were then washed again and 4-fold serial dilutions of guinea pig serum prepared in PBS-T with 5% skim milk, was added to duplicate wells and incubated at RT for 1 hr. After another thorough wash, plates were incubated with an HRP-conjugated goat anti-guinea pig IgG (H + L) secondary antibody (KPL) diluted 1:2000 in PBS-T with 5% skim milk, incubated for a further 1 hr at RT and again washed as above. Finally, plates were incubated in the dark for 30 min, RT with TMB solution (Life Technologies) after which the optical density was measured at 650nm (OD_650_) using a Synergy HTZ plate reader (Biotek). Cut-off values were established at 3X the standard deviation of the OD_650_ for samples collected from naïve guinea pigs.

**Cytokine analyses:** Serum samples were inactivated by gamma irradiation (5 mRad, Coblat-60 source) prior to analysis. Cytokine and chemokine responses were examined using a rat 27-plex panel (RECYMAG65K27PMX, Millipore Sigma) according to the manufacturer’s specifications. Serum samples were diluted 1:2 and test plates were run using a Luminex MAGPIX instrument. The analytes examined included EGF, CCL11 (Eotaxin), CX3CL1 (Fractalkine), G-CSF, GM-CSF, GRO/KC, IFN-γ, IL-1α, IL-1β, IL-2, IL-4, IL-5, IL-6, IL-10, IL-12 (p70), IL-13, IL-17A, IL-18, CXCL10 (IP-10), Leptin, LIX, MCP-1, MIP-1α, MIP-2, CCL5 (RANTES), TNF-α, VEGF. This kit has known cross-reactivity with guinea pigs samples [11]. An integrated cytokine score (CytoScore) from the linear combination of eight analytes (IL-1β, IL-2, IL-6, IL-10, IL-18, CCL5, CXCL10, and CX3CL1) was calculated for each sample as previously described [12].

**Serum biochemistry, hematology and coagulation parameters analysis:** Analysis of serum biochemistry was preformed using a VetScan VS2 analyzer (Abaxis Veterinary Diagnostics) using a comprehensive diagnostic profile reagent rotor per manufacturer instructions. Complete blood counts were performed on EDTA-treated cardiac blood using a VetScan HM5 hematology system (Abaxis Veterinary Diagnostics) per manufacturer instructions. Coagulation parameters including fibrinogen, activated partial thromboplastin time (aPTT), prothrombin time (PT) and Thrombin were analyzed on citrate treated plasma using Satellite Max instrument (Stago). Due to the volume of blood required, coagulation assessments were only done on exsanguinated animals.

**Data *a*nalysis:** Results were analyzed and graphed using GraphPad Prism version 10.5.0 (GraphPad Software, La Jolla, CA, USA). Every data set was tested for statistical normality and this information was used to apply the appropriate (parametric or nonparametric) statistical test. Time-to-death comparisons (global and pairwise) were done using the logrank test corrected for multiple comparisons with the Holm-Sidak method (Fig 4). Statistical significance between age groups were determined using a one-way ANOVA with a Holm-Sidak post-test or a Kruskal-Wallis test with a Dunn’s post-test (Figs 4–9). Statistical significance between sexes with each age groups were assessed using an unpaired t test or a Mann-Whitney U test (Fig 4), or using a two-way ANOVA with a Holm-Sidak post-test (Figs 5–9). p values < 0.05 were considered significant; significance values are indicated as *p < 0.05, **p < 0.01, ***p < 0.001, ****p < 0.0001.

**Fig 1:**
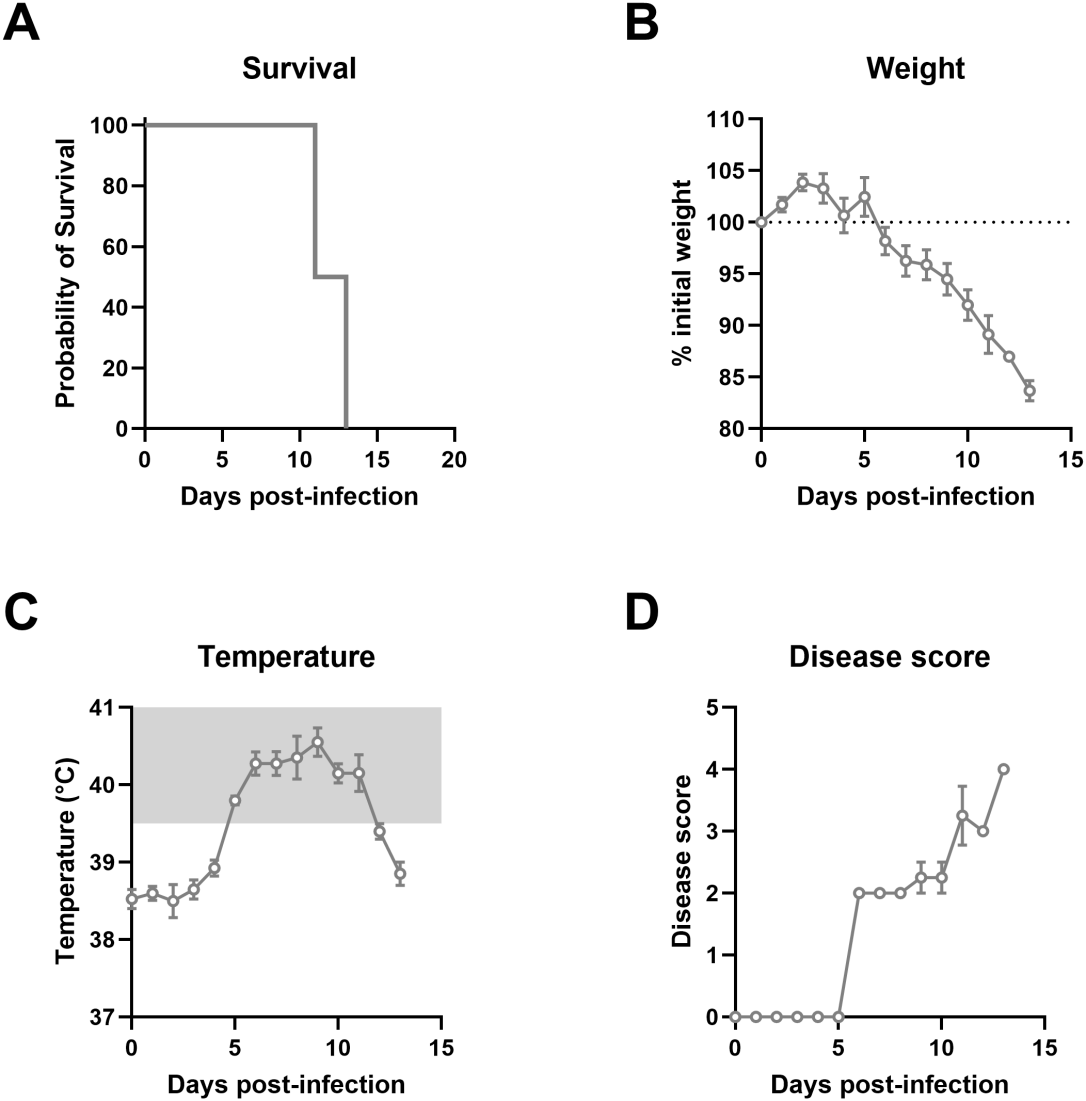
Disease monitoring of Lujo virus infected strain 13/N guinea pigs. Four adult (7-9 months old) strain 13/N guinea pigs were infected with 1x10^5^ TCID_50_ units of LUJV by the intraperitoneal (I.P) route. Animals were monitored for (A) survival, (B) weight loss, (C) body temperature, and (D) disease score. Data points and error bars represent group means ± SEM. Temperatures above normal ranges are indicated by gray shading.

**Fig 2:**
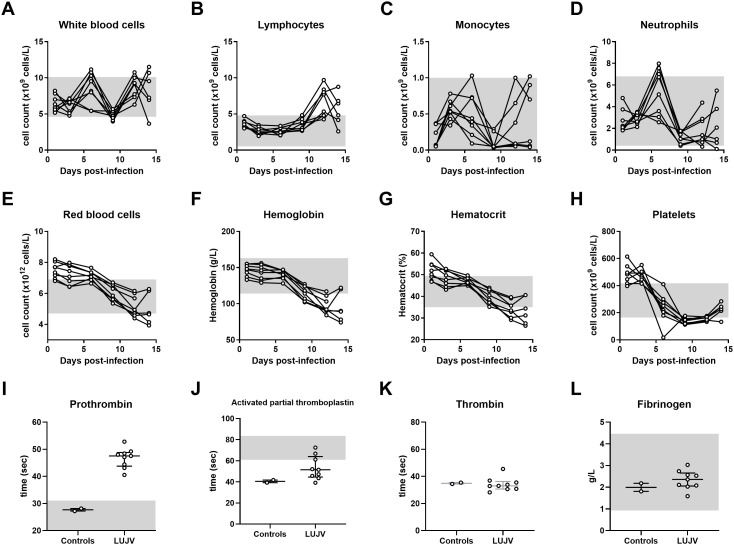
Time course assessment of hematological and coagulation parameters following Lujo virus infection in strain13/N guinea pigs. Juvenile strain 13/N guinea pigs were infected intraperitoneally with 1 × 10^5^ TCID₅₀ of LUJV. EDTA-treated whole blood samples (A-H) were collected at 1, 4, 6, 9, 12, and 14 days post-infection. A one-time, terminal citrate-treated plasma sample was collected at 14-16 DPI (I-L). Hematological parameters were measured with an HM5 hematology analyzer, and coagulation parameters with a Satellite Max analyzer. The following parameters were monitored: (A) white blood cells; (B) lymphocytes; (C) monocytes; (D) neutrophils; (E) red blood cells; (F) hemoglobin; (G) hematocrit; (H) platelets; (I) prothrombin; (J) activated partial thromboplastin; (K) thrombin; (L) fibrinogen. (A-H) Gray connecting lines indicate median values at each timepoint. (I-L) Data points and error bars represent group medians ± interquartile range (IQR).

**Fig 3:**
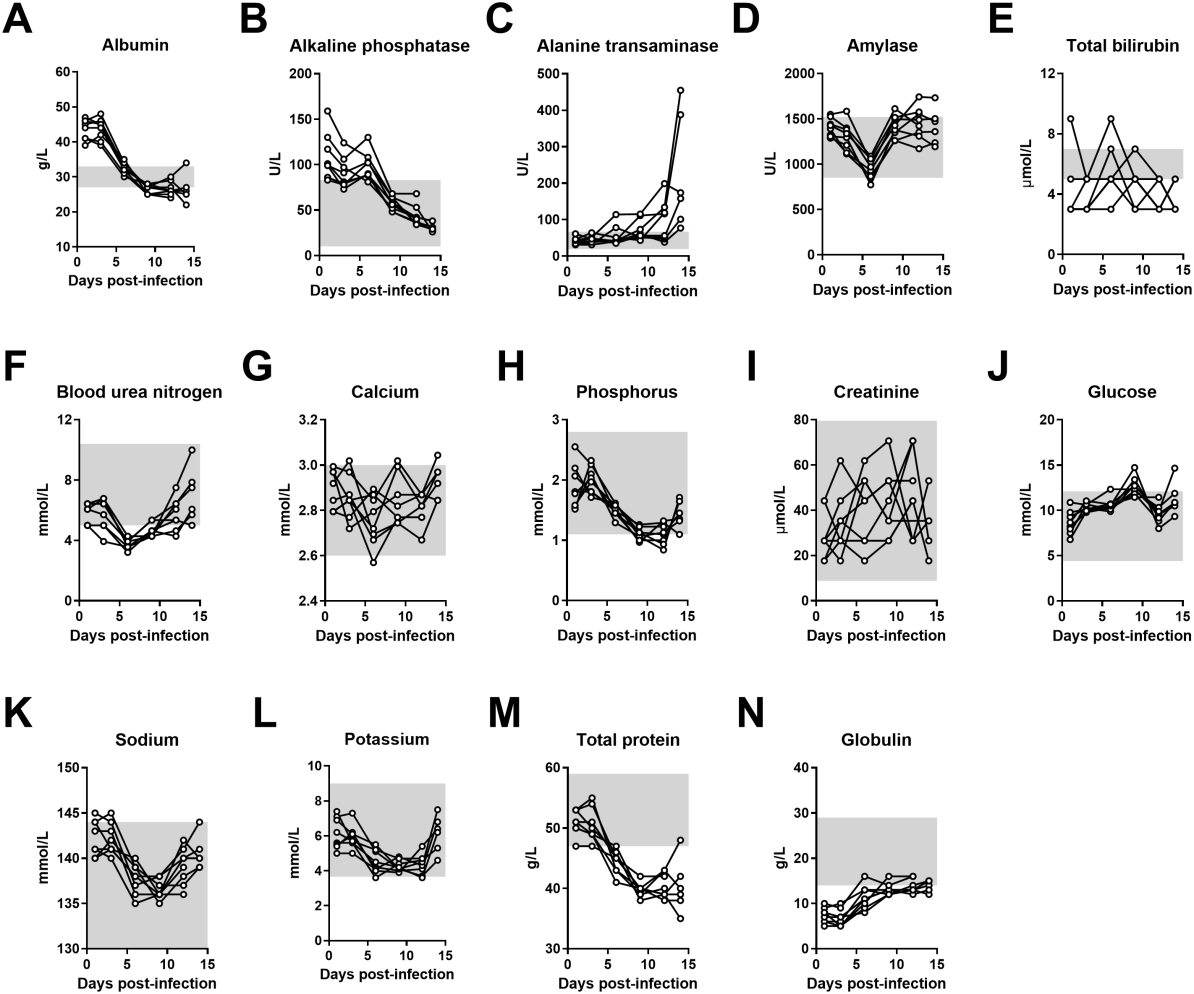
Time course assessment of biochemical parameters following Lujo virus infection in strain13/N guinea pigs. Juvenile strain 13/N guinea pigs were infected intraperitoneally with 1 × 10^5^ TCID₅₀ of LUJV. Serial serum samples (A-N) were collected at 1, 4, 6, 9, 12, and 14 days post-infection. Samples were monitored for levels of (A) albumin, (B) alkaline phosphatase, (C) alanine transaminase, (D) amylase, (E) total bilirubin, (F) blood urea nitrogen, (G) calcium, (H) phosphorus, (I) creatinine, (J) glucose, (K) sodium, (L) potassium, (M) total protein, and (N) globulin using a VS2 biochemistry analyzer. Gray connecting lines indicate median values at each timepoint.

**Fig 4:**
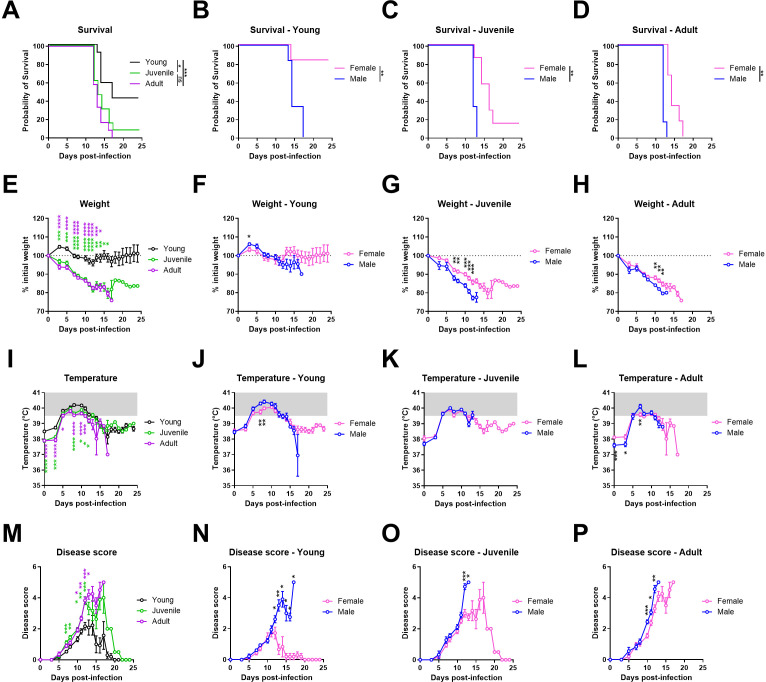
Age- and sex-dependant disease progression in Lujo virus infected strain 13/N guinea pigs. Young (1-2 months old), juvenile (3-6 months old), and adult (7-10 months old) strain 13/N guinea pigs were infected with 1x10^5^ TCID_50_ units of LUJV by the intraperitoneal (IP) route. Animals were monitored for (A-D) survival, (E-H) percent weight loss, (I-L) temperature, and (M-P) disease score. Each age group consists of 18-21 animals (both sexes), with 6-9 animals euthanized at 12 DPI. Data points and error bars represent group means ± SEM Temperatures above normal ranges are indicated by gray shading. The dotted lines depict the limit of detection of the assays. (A-D) Statistical significance for survival curves was assessed by logrank test. (E,I,M) Statistical significance between age groups was assessed by one-way ANOVA with a Holm-Sidak post-test or Kruskal-Wallis test. (F-H, J-L, N-P) Statistical significance between sex groups was assessed by unpaired t test or Mann-Whitney U test. *P < 0.05; **P < 0.01; ***P < 0.001; ****P < 0.0001; ns, not significant..

**Fig 5:**
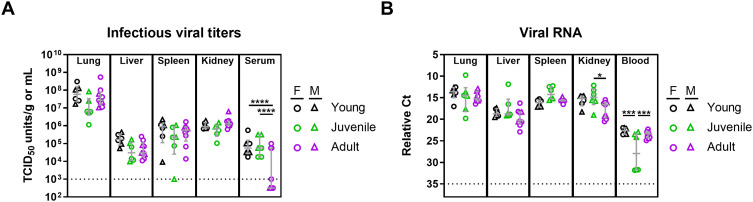
Lujo virus replication in strain 13/N guinea pigs. Male and female strain 13/N guinea pigs of three age groups - young (1-2 months old), juvenile (3-6 months old), and adult (7-10 months old) were infected with LUJV and euthanized at 12 days post-infection (DPI) for tissue and serum collection. (A) Infectious viral titers in tissues and serum were determined using the 50% tissue culture infectious dose (TCID_50_) assay. (B) Viral RNA levels in tissues and serum were quantified by RT-qPCR. Each age group consists of 6-9 animals (both sexes). Females and males are depicted as circles and triangles, respectively. Data points and error bars represent group medians ± interquartile range (IQR). Statistical significance was assessed by one-way ANOVA with a Holm-Sidak post-test or Kruskal-Wallis test with a Dunn’s post-test.*P < 0.05; ***P < 0.001; ****P < 0.0001. Ct, cycle threshold.

**Fig 6:**
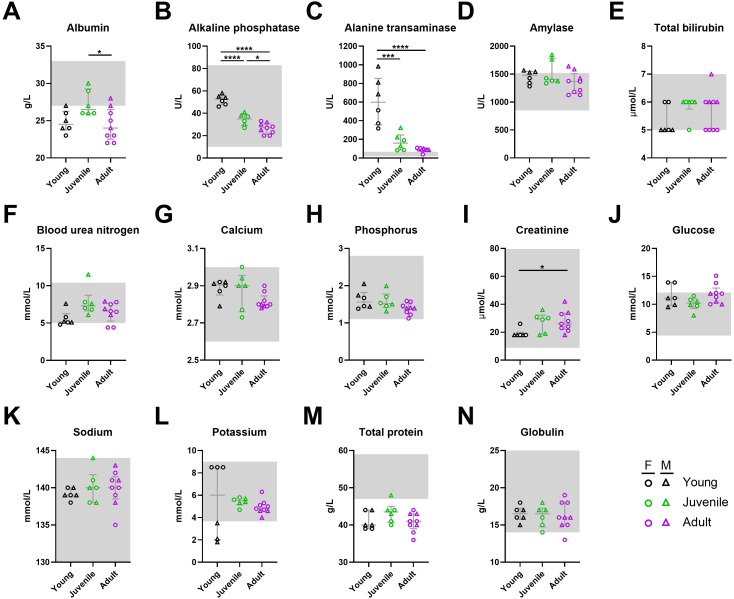
Blood biochemical parameters following Lujo virus infection in strain 13/N guinea pigs. Serum samples collected from LUJV-infected strain 13/N guinea pigs at 12 DPI were monitored for levels of (A) albumin, (B) alkaline phosphatase, (C) alanine aminotransferase, (D) amylase, (E) total bilirubin, (F) blood urea nitrogen, (G) calcium, (H) phosphorus, (I) creatinine, (J) glucose, (K) sodium, (L) potassium, (M) total protein, and (N) globulin using a VS2 biochemistry analyzer. Each age group consists of 6-9 animals (both sexes). Females and males are depicted as circles and triangles, respectively. Data points and error bars represent group medians ± interquartile range (IQR). Where available, normal ranges are indicated by gray shading [13,14]. Statistical significance was assessed by one-way ANOVA with a Holm-Sidak post-test or Kruskal-Wallis test with a Dunn’s post-test. *P < 0.05; ***P < 0.001; ****P < 0.0001.

**Fig 7:**
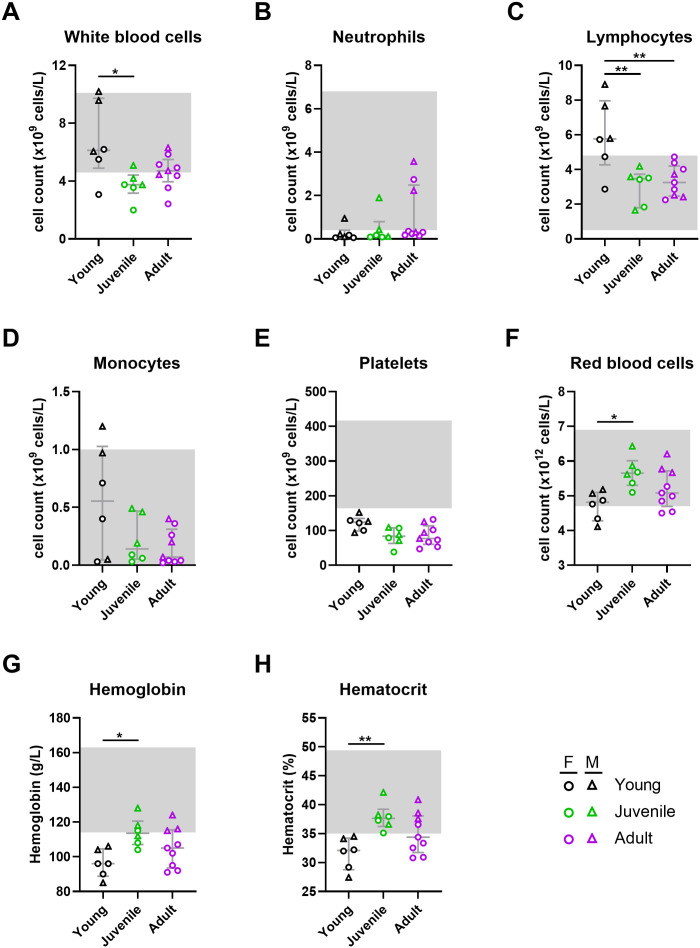
Hematological parameters following Lujo virus infection in strain 13/N guinea pigs. EDTA-treated whole blood samples collected from LUJV-infected strain 13/N guinea pigs at 12 DPI were analyzed using the HM5 hematology analyzer. The following parameters were monitored: (A) white blood cells, (B) neutrophils, (C) lymphocytes, (D) monocytes, (E) platelets, (F) red blood cells, (G) hemoglobin (HB), (H) hematocrit (HCT). Each age group consists of 6-9 animals (both sexes). Females and males are depicted as circles and triangles, respectively. Data points and error bars represent group medians ± interquartile range (IQR). Where available, normal ranges are indicated by gray shading area [13,14]. Statistical significance was assessed by one-way ANOVA with a Holm-Sidak post-test or Kruskal-Wallis test with a Dunn’s post-test. *P < 0.05; **P < 0.01.

**Fig 8:**
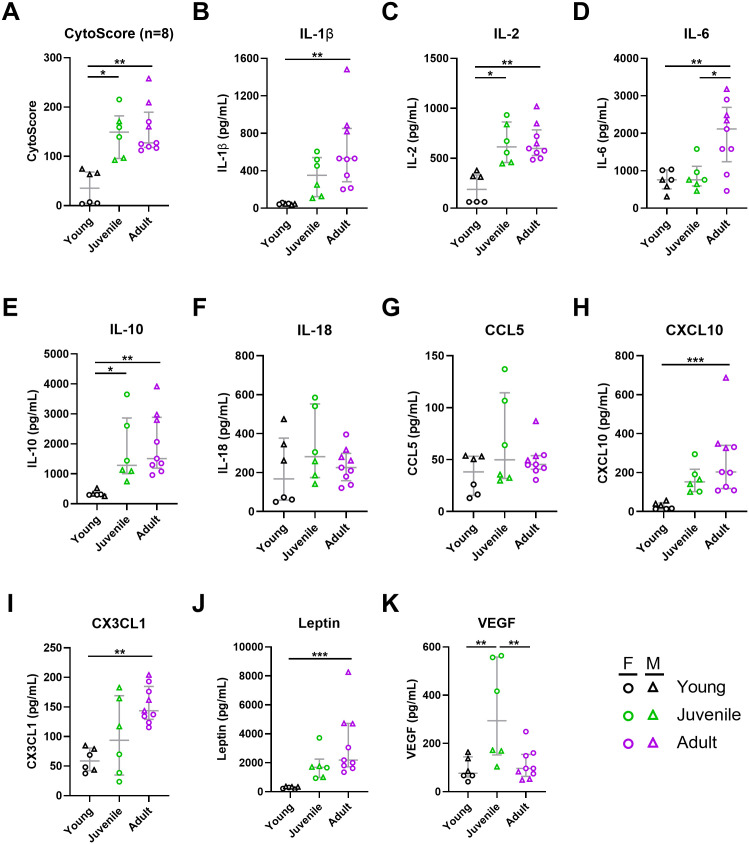
Host responses following Lujo virus infection in strain 13/N guinea pigs. Male and female strain 13/N guinea pigs of three age groups— young (1-2 months old), juvenile (3-6 months old), and adult (7-10 months old) were infected with LUJV and euthanized at 12 days post-infection (DPI) for assessment of host responses. (A) Cytokine scores (CytoScore) were calculated as the linear combination of 8 cytokines (IL-1β, IL-2, Il-6, IL-10, IL-18, CCL5, CXCL10, CX3CL1). Individual cytokines (B-I, and other inflammatory markers (J-K) were assessed by Luminex assay. Each age group consists of 6-9 animals (both sexes). Females and males are depicted as circles and triangles, respectively. Data points and error bars represent group medians ± interquartile range (IQR). Statistical significance was assessed by one-way ANOVA with a Holm-Sidak post-test or Kruskal-Wallis test with a Dunn’s post-test. *P < 0.05; **P < 0.01; ***P < 0.001.

**Fig 9:**
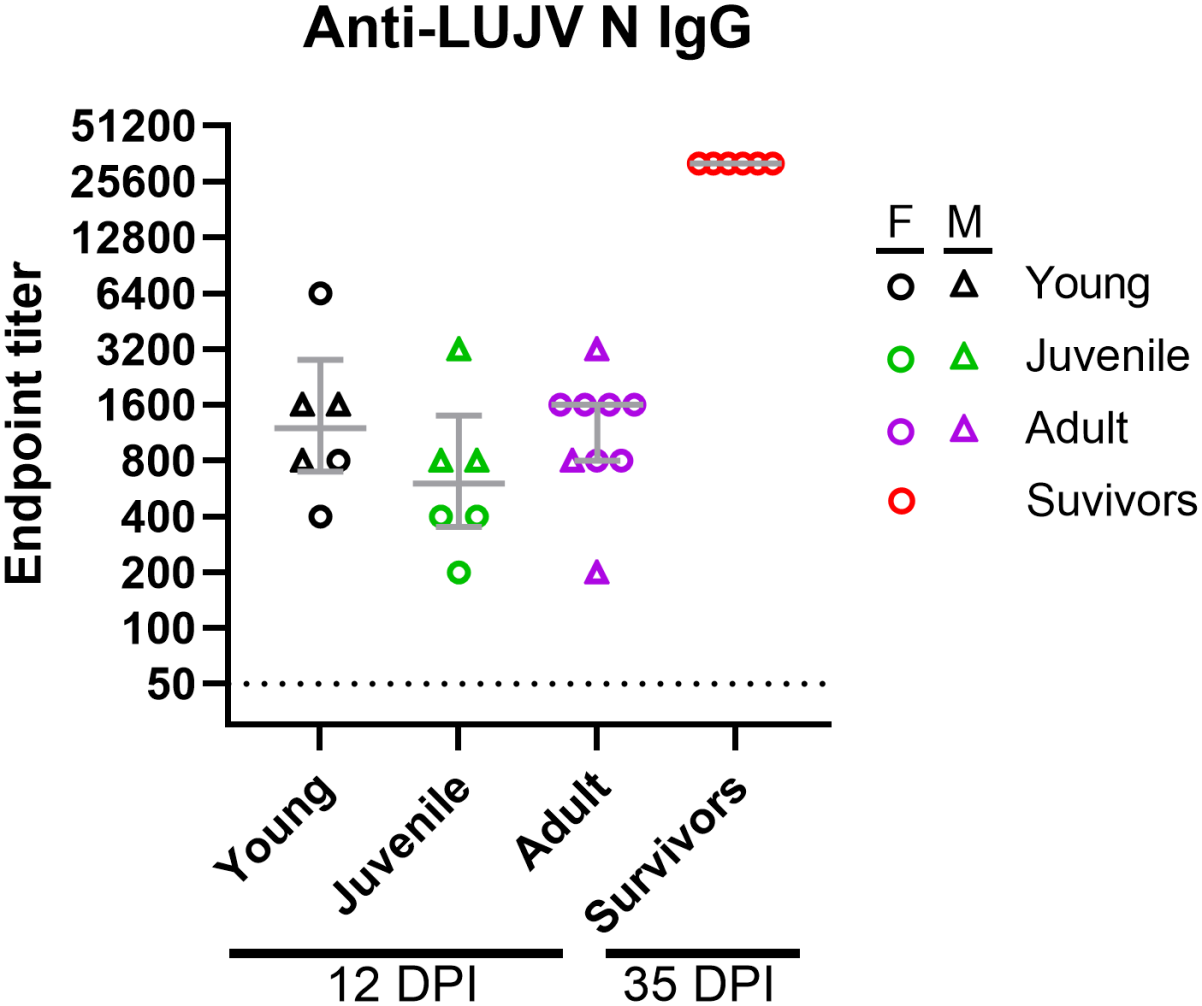
Humoral responses in strain 13/N guinea pigs following Lujo virus infection. Male and female strain 13/N guinea pigs of three age groups— young (1-2 months old), juvenile (3-6 months old), and adult (7-10 months old) were infected with LUJV and euthanized at 12 days post-infection. Serum samples were analyzed for the presence of anti-Lujo virus antibodies using a recombinant nucleocapsid (N) protein. Data shown represents reciprocal endpoint IgG titers as determined using standard ELISA methodologies. Each age group consists of 6-9 animals (both sexes). Survivors are all females. Females and males are depicted as circles and triangles, respectively. Data points and error bars represent group medians ± interquartile range (IQR). The dotted line depicts the limit of detection of the assay.

## Results

### LUJV infection in adult strain 13/N guinea pigs is lethal

In order to confirm lethality of LUJV infection in strain 13/N guinea pigs, 4 animals were inoculated via IP injection and monitored daily for signs of disease, including appearance, weight loss and temperature (Fig 1). Beginning at 6 DPI, signs of disease were apparent and included weight loss, increased respiration rate and elevated temperatures exceeding 40°C. Within 5–7 days post-symptom onset (11 and 13 DPI), disease severity in all four animals had progressed to the pre-determined endpoint requiring euthanasia. Terminal disease was characterized by prolonged elevated temperature reaching up to 41°C, laboured breathing, and weight loss between 15–18%. These findings mirrored the original description of the model, albeit in animals that were several months younger then those used by Bird et al. [7]. Based on our observations, we sought to examine disease progression in juvenile animals with an additional analysis of serial changes in hematological and biochemical parameters post- UJV infection. To that end, a group of 8 juvenile animals were inoculated as above and blood samples were serially collected every three days post-challenge over a two-week course (Figs 2 and 3). Gradual declines in red blood cell counts, hemoglobin, hematocrit and platelet counts were observed throughout the time-course study, though platelet levels showed a slight increase at 14 DPI (Fig 2E–H). Conversely, lymphocyte counts progressively increased until a slight drop at 14 DPI (Fig 2B). Monocytopenia and neutropenia was observed as noted by decreased populations at 9 DPI, with partial recovery in some animals by 14 DPI (Fig 2C,D). Liver dysfunction was evident by the steady decline in total protein, albumin, alkaline phosphatase levels (Fig 3A, B, M) throughout infection, alongside a notable increase in ALT (Fig 3C). Mild kidney dysfunction emerged at later stages, reflected by changes in BUN, and potassium levels (Fig 3F, K). All guinea pigs in this cohort reached the pre-determined endpoint based on disease severity and were euthanized between 14–16 DPI. At the time of euthanasia, cardiac blood was collected in citrate vacutainer for a one-time analysis of coagulation factors in fresh plasma. Notably, PT indices increased from 27-28 s (average 27.7 s) in naïve guinea pigs to 40–49 s (average 46.1 s) in terminally ill animals (Fig 2I). Similarly, aPTT increased from between 39–41 (average 40.5 s) seconds to between 39 and 66 seconds (average 49 s) (Fig 2J). No change in Thrombin time was observed (34.6 in naïve animals versus 34.2 in terminally ill) (Fig 2K). A slight increase in Fibrinogen was noted (2.0 g/L in naïve animals versus 2.5 g/L in terminally ill animals) (Fig 2L).

### LUJV pathogenicity is influenced by age and sex in strain 13/N guinea pigs

Although the original description of LUJV infection and disease in strain 13 guinea pigs utilized animals ranging from 1-1.5 years of age, the results of our first experiments demonstrated that younger animals were also susceptible to lethal disease. To further address this, we sought to refine the model and assess disease progression in animals of differing ages and of both sexes. To that end, groups of 18 guinea pigs (9 male, 9 female) were categorized as young (1–2 months old), juvenile (3–6 months old old) or adult (7–10 months old) were challenged with LUJV and disease progression was monitored (Fig 4, Tables 1 and S1). Clinical signs began to appear at 5 DPI, with disease scores peaking at 12 DPI for all age groups (Fig 4M–P). Young animals, particularly young females, exhibited a lower peak disease score compared to juvenile and adult animals of either sex. All infected guinea pigs displayed ruffled fur, while juvenile and adult animals presented with increased respiratory rate, lethargy, and signs of ocular distress. Ataxia was observed in one juvenile male and three adult male guinea pigs, but it was absent in young animals and in all females. Across all groups, body temperatures peaked between 39.6°C and 41.1°C, with no significant differences between groups (Fig 4I–L). Elevated body temperatures were observed from 5 to 12 DPI. Body temperatures in animals which survived LUJV infection returned to baseline by 15 DPI, while those which progressed to terminal disease had a precipitous drop in temperature immediately preceding death. Juvenile and adult guinea pigs experienced a marked reduction in food and water intake starting at 3 DPI, leading to progressive weight loss until they met the pre-determined endpoint criteria (Fig 4E–H). Animals in these groups lost approximately 20% of their starting weight during the course of LUJV infection. Weight loss was slightly delayed in female animals which was consistent with increased time to lethal disease. All juvenile and adult animals, except one juvenile female, reached endpoint criteria by 18 DPI (Fig 4A–D). In contrast, young female animals resisted weight loss, with initial weight gain observed before stabilizing at baseline levels by 7 DPI (Fig 4B, F). Notably, 5/6 young female guinea pigs survived, while all young males succumbed to the infection by 17 DPI.

**Table 1:** Clinical signs and disease progression of Lujo virus infected strain 13/N guinea pigs. ↑ RR = Increase respiratory rate; S = survivors, L = lethal; * = 5/6 survivors; ** = 1/6 survivors.

		Clinical signs			Disease progression			
Group	Sex	0-4 (DPI)	5-10 (DPI)	11-Terminal (DPI)	Time of Death (DPI)	Peak Disease score	Peak Temperature	Peak weight Change
					Median [range]	Median [range]	Range	Range
Young (1–2 months old)	Male	Normal	↑ RR	↑ RR, ruffled fur, respiratory distress	14 [13-17]	5	40.1-40.9 °C	-14.7% to +2.7%
	Female	Normal	↑ RR	S: Normal, ruffled fur L: Respiratory distress	14*	S: 2 [0–2]* L: 4	S: 39.9-40.3 °C L: 40.4 °C	S: –17% to +9.1%. L: + 6.9%
Juvenile (3–6 months old)	Male	Normal	↑ RR	Ruffled fur, ↑ RR, respiratory distress, hunched posture, ataxic, balance issues, ocular distress	12 [12-13]	5	39.9-40.3 °C	-19.8% to ≥ -20%
	Female	Normal	↑ RR	S: Ruffled fur, ↑ RR L:Ruffled fur, ↑ RR, respiratory distress, ocular distress	14 [12-17]**	S: 2.5** L: 5	S: 40-40.4 °C L: 40.4 °C	S: -16.3% L: -16% to ≥ -20%
Old (7–10 months old)	Male	Normal	↑ RR	Ruffled fur, ↑ RR, labored breathing, respiratory distress, ocular distress	12 [12-13]	5	39.3-40.5 °C	-18% to ≥ -20%
	Female	Normal	↑ RR	Ruffled fur, ↑ RR, respiratory distress, labored breathing, ocular distress	14 [12-17]	5	39.6-40.2 °C	-14.9% to ≥ -20%

At 12 DPI, a time corresponding to peak disease manifestations and immediately preceding death, at least 6 animals per age grouping (3 males, 3–6 females) were euthanized for the purpose of assessing viral burdens as well as a comparative analysis of serum biochemistries and hematology. High viral burdens were noted in lung, liver, spleen, kidney and serum samples from this cohort of animals by molecular and/or infectious assays (Fig 5A, B, S1 Table). Viral RNA was consistently detected in all tissues analyzed. Median infectious titers ranged from 10^5^ to 10^8^ infectious units per g or mL of tissues, with the highest titers observed in lungs (Fig 5A). Overall, viral burden was not influenced by age or sex of the animals.

At 12 DPI, age-related trends were apparent for some blood chemistry markers (Fig 6, S1 Table). Alanine aminotransferase (ALT) levels above normal values were observed across all age groups, but were particularly elevated in young guinea pigs compared to juvenile and adult animals, potentially indicating increased liver stress in younger individuals (Fig 6C). In contrast creatinine and blood urea nitrogen (BUN) levels slightly increased with age, with adult guinea pigs displaying higher concentrations than younger ones, which may suggest age-related declines in kidney function and increased tissue breakdown, however age related increases could be evident [13,14] (Fig 6F). Although age-related differences were not observed in total protein and albumin levels, values across the three age-groups were below normal values suggesting LUJV resulted in vascular leakage (Fig 6A, M). Only marginal differences between sexes were observed within the same age group, including higher ALT (p < 0.0001) and lower potassium (p < 0.0001) in young males as compared to young females (Fig 6C,L), which could have contributed to the marked difference in disease outcome within the younger age group (Fig 4B).

At 12 DPI, most hematological parameters monitored were within or near normal ranges (Fig 7, S1 Table). Total white blood cells (WBC) counts, most notable lymphocytes were slightly higher in young animals compared with juveniles and adults (Fig 7A–D). The most striking findings was platelet counts which at 12 DPI were below normal range in all three age groups and indicative of thrombocytopenia (Fig 7E). Hemoglobin levels were lowest in young guinea pigs, though average values in all three age groups were below normal levels (Fig 7G). Similarly, hematocrit was lowest in young animals and also below normal ranges in young and adult animals, but within range in juveniles (Fig 7H). Average red blood cell counts were within normal range in all age groups but followed a similar age-related trend as hemoglobin (Fig 7F). Sex-related differences were observed in young animals where males displayed significantly higher counts of WBC (p = 0.0140) and lymphocytes (p = 0.0186) as compared to females, indicative of a stronger immune activation in young males.

### Modulation of immune responses in strain 13/N guinea pigs is influenced by age and sex

The variation in guinea pig survival outcomes during LUJV challenge prompted us to explore the host immune responses, more specifically cytokine and antibody responses. The expression of cytokines, chemokines, and other inflammatory markers was quantified in serum samples collected at 12 DPI using a rat 27-plex Luminex panel, though cross-reactivity was observed in only 10 out of 27 analytes tested. These included IL-1β, IL-2, IL-6, IL-10, IL-18, CCL5 (RANTES), CXCL10 (IP-10), CX3CL1 (Fractalkine), leptin, and VEGF. There were striking differences between sex and age groups in the overall cytokine responses, as calculated using an integrated cytokine score (CytoScore) [12] (Fig 8A). Juvenile and adult animals (average CytoScore 145.6 and 155.4, respectively) displayed higher levels of cytokines as compared to the young animals (average CytoScore 36.6). Individual cytokines and inflammatory markers were also largely influenced by age, as levels of IL-1β, Il-2, IL-6, IL-10, CXCL10, CX3CL1 and leptin were all their lowest in younger animals (Fig 8B–K, S1 Table). Sex-related differences were also evident, with males exhibiting a stronger cytokine storm than age-matched females, particularly in the young group. Among the young group, females who survived challenge demonstrated the greatest disparity compared to age-matched males. Indeed, cytokine responses in young females (average Cytoscore 5.2) were 13-fold lower as compared to young males (average Cytoscore 68.1), and this difference was found to be significant (p = 0.0469) (Fig 8A). Young females exhibited a weakest cytokine response for most analytes, showing up to 5-fold reduction in IL-2, IL-18, CCL5, and CXCL10 as compared to their male counterpart (Fig 8B–K). Surprisingly, only one cytokine was found to be significantly induced in young females when compared to uninfected controls (IL-6, 1.2-fold increase), highlighting the absence of a cytokine storm in this subset of animals. This strongly suggest a negative correlation between the magnitude of the pro-inflammatory responses and the probability of survival.

Anti-LUJV humoral responses were measured in guinea pigs using an in-house recombinant LUJV N ELISA. Serum samples collected from animals as a part of the 12 DPI necropsy time point yielded similar IgG responses with endpoint reciprocal titers ranging from 200 to 6400. Median endpoint titers were similar across the three age groups and did not differ between sexes (Fig 9). Animals that survived LUJV infection all seroconverted to the LUJV N antigen with increased levels of specific IgG antibodies (end-point reciprocal titers of 25,600), as measured at 35 DPI.

## Discussion

Like many pathogenic arenaviruses, guinea pigs are an important small animal model for the study of LUJV infection and ensuing disease, as well as a means to evaluate potential vaccines and therapeutics [15–17]. With respect to LUJV, inbred guinea pigs, remain the only animal model described to date suitable for these purposes. Although strain 13/N guinea pigs infected with LUJV recapitulate many aspects of VHF-like disease, the previous description of the model only utilized animals greater then 1 year of age [7]. Strain 13/N guinea pigs are not readily commercially available, meaning the use of these animals for disease modelling requires access to in-house breeding colonies. Acquiring sufficient numbers of guinea pigs aged 1 year or older to conduct medical countermeasures evaluations or pathogenicity studies would be logistically and financially challenging for many breeding colonies. The intent of the current study was to expand upon the original findings and assess LUJV infection and disease progression in the same inbred guinea pig model using animals of both sexes and varying ages.

Our results suggest that both age and sex influence LUJV pathogenesis in the inbred guinea pig model (Fig 4). Clinically, LUJV infection in inbred guinea pigs presented in both sexes and all age groups with non-descript signs of infection including hunched posture, ruffled fur, lethargy and inappetence beginning around 5 DPI. In general, disease severity in females was slightly delayed to that in age-matched males. LUJV infection in male guinea pigs was uniformly lethal with time to terminal disease ranging from 12 to 16 DPI. In contrast, lethal disease in age-matched females occurred 2–4 days later and was not uniformly lethal (Fig 4A–D). The delayed progression was not reflected in disease scores, which were primarily based on appearance and activity levels of individual animals, but was apparent in weight loss, which was more gradual in female animals (Fig 4E–H, M–P). A more profound impact of age was noted in the current study, with younger animals appearing less susceptible to lethal disease then juvenile and adult animals (Fig 4B–D). These differences were clear based on both disease scores observed between the age groupings as well as weight loss, with young animals scoring the lowest and losing the least amount of weight, even at the terminal stages of disease, when compared to their aged counterparts (Fig 4E–H, M–P). It should be noted though that young guinea pigs generally gain 5-7g in body weight each day until the age of 8 weeks old meaning that assessing disease progression in young guinea pigs based on minimal weight loss can be complicated [18]. Interestingly, body temperature profiles for both male and female animals of all age groups essentially mirrored one another, with temperatures nearing or exceeding 40°C in all infected animals beginning by 7 DPI and continuing until 15 DPI (Fig 4I–L). A precipitous drop in body temperature was observed in terminally ill animals immediately preceding euthanasia.

In order to assess infection kinetics and host responses in LUJV-infected animals, at least 3 male and 3 female animals per age group were euthanized at 12 DPI, a time corresponding to peak disease in most animals and when terminal disease was first apparent, for in-depth analysis including assessment of infectious titers as well as blood hematology and biochemistries, and serum cytokine/chemokine and antibody responses. Despite differences in disease manifestations, molecular and infectious assays revealed similar viral burdens in specimens collected from males and females across all age groups (Fig 5A,B). Lungs had the highest viral burden with average titers ranging from 10^6^ to 10^8^ infectious unit/g while titers in liver, spleen, kidney and serum samples averaged between 10^4^ and 10^6^ per g or mL (Fig 5A). Haematologically, LUJV-infected guinea pigs of both sexes and ages demonstrated similar profiles, with subtle variations, though most values remained within the normal range (Fig 7). Decreased platelet counts was a hallmark of disease in all animals (Fig 7E). Similarly, neutrophil levels were below normal values in most animals (Fig 7B). Of the few notable differences between age groups, leukocyte counts in young animals were elevated compared to juvenile and adult animals which was primarily driven by lymphocytes and monocytes. Biochemical trends were also mostly consistent across the age groups with trends of decreased serum albumin and total protein, and increased ALT suggesting that all LUJV-infected animals had increased vascular permeability as well as liver dysfunction (Fig 6A, C, M). Although still within normal ranges, BUN levels and creatinine levels in juvenile and adult animals were elevated when compared to younger animals suggesting some degree of kidney disease as well (Fig 6F, I). Conversely, ALP levels in LUJV-infected guinea pigs decreased with age which may be indicative of age related disease differences, though it could also be caused by malnutrition associated with lack of weigh gain in young and developing animals (Fig 6B). The above noted variations between age groups may also be influenced by sex. For example, white blood cell and lymphocyte counts (Fig 7A, C) as well as ALT levels appear to differ between the sexes, at least in the young age grouping. Young animals, particularly female survivors, exhibited higher ALT levels but maintained other indicators of hepatic function, including TBIL, at comparatively stable levels. This pattern suggests that the ALT rise in these animals represented an acute, regulated stress response rather than progressive liver failure. Overall trends observed in these animals confirmed the findings observed in the animals serially monitored. These differential factors could help explain why younger females show improved survival, but further study is required to better elucidate these potential patterns.

The most striking difference between both age and sex noted in the current study was in host responses. Cytokine profiling revealed that immune activation increased with age and was more pronounced in males (Fig 8). Adult males mounted the most robust cytokine responses, with elevated levels of pro-inflammatory mediators such as IL-1β, Il-2, IL-6, IL-10, CXCL10, and leptin(Fig 8 B–D, H, J). Notably, CXCL10 levels in adult males were over 30-fold higher than in young females, indicative of a strong cytokine storm that may have contributed to lethality (Fig 8H). In contrast, young females displayed the lowest cytokine responses (Fig 8A), particularly in IL-2, IL-18, CCL5, and CXCL10 (Fig 8C, F–H). The attenuated cytokine response when compared to other groups, suggests that females in the young age group mounted a more regulated immune response, which may have improved outcome to infection. In support of this, previous studies in NHPs comparing immune profiles from lethal LASV infections to non-lethal LUJV infection suggested postponed transcriptional responses of specific host genes contributed to the milder pathogenic phenotype and survival in that model [6].

Further time-course experiments are required to assess temporal changes in the host immune responses to LUJV infection and the potential role they play in pathogenesis. Previously, tissue-specific transcriptional downregulation and/or activation of selected host gene responses were observed over the course of LUJV infection in strain 13/N guinea pigs [7]. In that study, the authors speculate that although immune-mediated pathology may have contributed to organ damage, the complete dysregulation of the host immune response was not responsible for vascular permeability observed in the model. Our data also supports this hypothesis in that markers of vascular permeability, including serum albumin and total protein levels, were similarly reduced in all LUJV-infected animals despite the aforementioned age-specific differences in host responses. Nevertheless, In the current study, age- and sex- related differences in host response did appear to contribute to disease severity and progression to lethal disease. Although only a single time point was monitored in the current study, at the peak of disease manifestation and despite similarities in viral replication, young female animals exhibited lower overall cytokine scores and had increased survivors compared to males of all age groups as well as older females. Specifically comparing responses in young males, in which LUJV infection was lethal, to young females, who mostly resisted lethal disease, differences in IL-2, IL-18, CCL5, and CXCL10 standout. For these three analytes young males demonstrated higher values then young females with concentrations in the former group better aligned with values from older male and female guinea pigs in which LUJV infection was lethal. Based on these findings, we speculate that the role that IL-2, IL-18, CCL5, and CXCL10 have on inflammation and activation of natural killer and cytotoxic T cells may be pivotal in LUJV pathogenesis. However, to fully define the roles these cytokines and chemokine have on LUJV further studies are required with large groups of animals.

Despite having only one documented outbreak consisting of 5 confirmed cases, the public health importance of LUJV as an emerging pathogen should not be underestimated. In addition to clinical surveillance for suspected cases across Africa, laboratory preparedness efforts, including the development of animal models to both study pathogenesis of disease as well pre-clinical characterization of potential antivirals and vaccines, should continue in order to minimize future spillover events. The results of this current study provide important refinement and characterization data to the previously described strain 13/N guinea pig model. Specifically, our findings highlight the influence of age and sex on the pathogenesis of LUJV in strain 13/N guinea pigs. Although equally susceptible to LUJV, young female guinea pigs demonstrated relative resistance to lethal disease with infection characterized by lower cytokine responses and improved survival rates compared to sex-matched juvenile and adult animals. In contrast, male guinea pigs of all ages were more susceptible to lethal disease which was associated with greater immune activation and cytokine scores. These results build on previous descriptions of this model and will help advance both pathophysiology studies for LUJV disease as well as serve as a more convenient animal model for medical countermeasures assessments.

## Supporting information

S1 Table Sex-related differences for LUJV-infected strain 13/N guinea pigs for virological, biochemical, hematological, and immunological parameters per age group. (DOCX)

S1 Data Excell spreadsheet containing raw data from all experimental analysis conducted in this study. (XLSX)
