# Supplementary material for: Age- and sex-associated differences in Lujo hemorrhagic fever pathogenesis in strain 13/N guinea pigs
Source: PLoS Negl Trop Dis. 2025 Oct 21;19(10):e0013633. doi: 10.1371/journal.pntd.0013633 (PMC12551956; doi:10.1371/journal.pntd.0013633)
Supplement: S1 Table — (DOCX) [file pntd.0013633.s001.docx]

**Table S1. Sex-related differences for LUJV-infected strain 13/N guinea pigs for virological, biochemical, hematological, and immunological parameters per age group**

| Parameters | | Statistical significance (adjusted p value)* | | |
| --- | --- | --- | --- | --- |
|  |  | Young (1-2 months) | Juvenile (3-6 months) | Adult (7-10 months) |
| Viral burden | Infectious titers - Lung | 0.9954 | 0.8074 | 0.9952 |
|  | Infectious titers - Liver | 0.9952 | 0.9944 | 0.9954 |
|  | Infectious titers - Spleen | 0.9952 | 0.9592 | 0.9391 |
|  | Infectious titers - Kidney | 0.9954 | 0.9592 | 0.9952 |
|  | Infectious titers - Serum | 0.9952 | 0.8009 | 0.0906 |
|  | Viral RNA - Lung | 0.9947 | 0.9984 | 0.9807 |
|  | Viral RNA - Liver | 0.9998 | 0.2258 | 0.9998 |
|  | Viral RNA - Spleen | 0.9807 | 0.4663 | 0.9984 |
|  | Viral RNA - Kidney | 0.9998 | 0.1280 | 0.9984 |
|  | Viral RNA - Blood | 0.9998 | **<0.0001** | 0.9998 |
| Serum biochemistry | Albumin (ALB) | 0.7458 | 0.7458 | 0.1079 |
|  | Alkaline phosphatase (ALP) | 0.1706 | 0.4213 | 0.4213 |
|  | Alanine transaminase (ALT) | **<0.0001** | 0.2296 | 0.9092 |
|  | Amylase (AMY) | 0.0726 | **0.0280** | **0.0072** |
|  | Total bilirubin (TBIL) | 0.1968 | 0.4001 | **0.0267** |
|  | Blood urea nitrogen (BUN) | 0.7271 | 0.7114 | 0.7271 |
|  | Calcium (CA) | 0.6715 | 0.4047 | 0.6464 |
|  | Phosphorus (PHOS) | 0.4637 | 0.9688 | 0.9688 |
|  | Creatinine (CRE) | 0.6240 | 0.5812 | 0.5812 |
|  | Glucose (GLU) | 0.0842 | 0.3169 | 0.2661 |
|  | Sodium (NA) | 0.8507 | 0.8345 | 0.8345 |
|  | Potassium (K) | **<0.0001** | 0.8230 | 0.1027 |
|  | Total protein (TP) | 0.7488 | 0.3272 | 0.4762 |
|  | Globulin (GLOB) | 0.6645 | 0.2022 | 0.6645 |
| Hematology | White blood cells (WBC) | **0.0140** | 0.4864 | 0.4864 |
|  | Neutrophils (NEU) | 0.6745 | 0.6011 | 0.1507 |
|  | Lymphocytes (LYM) | **0.0186** | 0.8243 | 0.6529 |
|  | Monocytes (MON) | 0.3609 | 0.3609 | 0.6289 |
|  | Platelets (PLT) | 0.7786 | 0.7786 | 0.7056 |
|  | Red blood cells (RBC) | 0.6679 | 0.1269 | **0.0041** |
|  | Hemoglobin (HGB) | 0.4707 | 0.1863 | **0.0044** |
|  | Hematocrit (HCT) | 0.6558 | 0.4815 | **0.0105** |
| Immunology | CytoScore | **0.0469** | 0.0581 | **0.0061** |
|  | IL-1β | 0.9552 | **0.0340** | **0.0001** |
|  | IL-2 | 0.0541 | 0.2357 | **0.0446** |
|  | IL-6 | 0.5238 | 0.5238 | **0.0469** |
|  | IL-10 | 0.8772 | **0.0042** | **0.0004** |
|  | IL-18 | **0.0035** | **0.0037** | 0.2679 |
|  | CCL5 | **0.0437** | **0.0003** | 0.0950 |
|  | CXCL10 | 0.7192 | 0.6998 | **0.0033** |
|  | CX3CL1 | 0.4504 | **0.0006** | 0.4480 |
|  | Leptin | 0.8802 | 0.7064 | **0.0002** |
|  | VEGF | 0.1216 | **<0.0001** | 0.1132 |
|  | Anti-LUJV N IgG | 0.6715 | 0.6715 | 0.9499 |

*Statistical significance was evaluated using two-way ANOVA with a Holm-Sidak post-test. Significant p values are highlighted in **bold**.
